# Anti-glutamate Dehydrogenase Antibody Positive Cerebellar Ataxia and Stiff Person Syndrome Responding to Dual Treatment with Steroids and Intravenous Immunoglobulin: A Case Presentation and Literature Review

**DOI:** 10.7759/cureus.4851

**Published:** 2019-06-06

**Authors:** Furkan M Yilmaz, Dena Little, Micheal Gallagher, Amy Colcher

**Affiliations:** 1 Neurology, Cooper Neurological Institute, Cooper University Hospital, Camden, USA; 2 Neurophysiology, University of Maryland Medical Center, Baltimore, USA

**Keywords:** hypothyroidism, cerebellar ataxia, steroids, intravenous immunoglobulins, scale for the assessment and rating of ataxia (sara), methylprednisolone, type 2 diabetes mellitus, glutamic acid decarboxylase antibody, stiff-person syndrome

## Abstract

Anti-glutamic acid decarboxylase (anti-GAD) antibody syndrome (aGAS) has various presentations including cerebellar ataxia (CA) and stiff person syndrome (SPS). This is a treatable cause of CA and SPS. We present a case of a 49-year-old man who developed blurred vision, slurred speech, difficulty walking, unsteady gait, and clumsiness which had progressed over four months. The patient was found to have anti-GAD ab (+) CA and SPS and experienced significant symptomatic improvements after treatment with intravenous (IV) steroids followed by intravenous immunoglobulin (IVIG). The patient’s improvement persisted when he was reevaluated at follow up one month later. Since anti-GAD ab related diseases, including anti-GAD CA and SPS, are rarely diagnosed, there is limited data regarding the treatment of this condition. As there are only a few cases in the literature similar to this one, highlighting the successful treatment of anti-GAD ab cerebellar ataxia and SPS with dual therapy (steroids followed by IVIG) is important.

## Introduction

Glutamic acid decarboxylase (GAD) is an enzyme which catalyzes the conversion of glutamate to gamma-aminobutyric acid (GABA). GAD is expressed in the central nervous system (CNS), pancreas, and thyroid gland [1]. GAD is an intracellular enzyme [2]. The mechanism of cell destruction with an extracellular antibody, and the role of the antibody in the pathophysiology of the disease, are not clear [3]. Animal studies give promising evidence for a possible mechanism of the disease [4]. Anti-GAD antibody (Ab) cerebellar ataxia is a rare cause of subacute, gait-predominant ataxia [5]. Most of the affected patients are women, many of whom also have Type 1 diabetes mellitus (DM) [3]. The diagnosis is made by the presence of elevated anti-GAD antibody levels in the serum or cerebrospinal fluid (CSF) [6]. Currently, there is limited data, primarily in the form of case reports, regarding the treatment of this condition. 

## Case presentation

A 49-year-old plumber presented to the hospital with blurred vision, slurred speech, difficulty walking, unsteady gait, and clumsiness which had progressed over four months. He suffered a fall while at work. He had back pain and noticed imbalance and difficulty with his gait. 

On examination, he was alert and oriented with mild pseudo-bulbar effect. Extra-ocular movements displayed saccadic overshoot. His speech was dysarthric and slow. Cranial nerves were intact. There was increased tone in the thoracolumbar paraspinal muscles. Deep tendon reflexes were 1+, and plantar responses were flexor bilaterally. There was dysdiadochokinesia, and all extremities demonstrated dysmetria. The patient had postural instability with a positive Romberg sign. His gait was wide-based and ataxic, and he was unable to ambulate more than a few steps due to imbalance. His score on the scale for the assessment and rating of ataxia (SARA) [7] was 12.

Basic labs as well as vitamin b12, angiotensin-converting enzyme level, antineutrophil cytoplasmic antibodies, myeloperoxidase antibodies, proteinase 3b antibodies, vitamin E level, ceruloplasmin, HIV 1 and 2, celiac disease panel, inflammatory markers (erythrocyte sedimentation rate (ESR) and c-reactive protein (CRP)), rheumatologic antibodies, and heavy metal screen were negative. Cerebrospinal fluid (CSF) analysis (cell count, protein, and glucose) were within normal limits. CSF oligoclonal band and CSF serology were negative. Paraneoplastic antibody testing including anti-Hu, anti-Yo, anti-Ri, anti-Ma was negative. CSF anti-GAD was positive with a titer of >250 international units per milliliter (IU/Ml). Magnetic resonance imaging (MRI) of the brain did not show an acute or chronic disease process. Computed tomography scan (CT) of the chest, abdomen, and pelvis was negative for any underlying malignancy. Prostate-specific antigen (PSA) was negative.

Intravenous methylprednisolone (500 mg twice a day) was initiated. The patient’s back pain significantly improved within 24 hours of the five-day course. By the end of the steroid course, his appendicular ataxia improved; however, his gait remained ataxic. Therefore, we initiated additional treatment with IVIG (2 mg per kg divided into a five-day course). By day five of the IVIG course, the patient’s ataxic gait significantly improved, and he was able to ambulate beyond 100 feet. By the day of discharge, his SARA score had improved to eight. Pre- and post-treatment videos of his neurological examination were taken. One month after the treatment, the patient was able to walk at least 100 feet without the help of a cane or walker.

## Discussion

GAD catalyzes the conversion of glutamate to GABA, and antibodies to this enzyme can lead to a multitude of neurologic syndromes including, but not limited to, limbic encephalitis, stiff person syndrome, refractory epilepsy, and cerebellar ataxia [8-9]. Anti-GAD antibody syndrome (aGAS) can present with thyroiditis, pernicious anemia, vitiligo, and type one diabetes [10-12]. In addition to hypothyroidism, our patient was diagnosed with type 2 diabetes mellitus, shortly before the diagnosis of aGAS. This may raise a question regarding the role of anti-GAD antibodies in insulin resistance as well [13]. A thorough neurological exam should be done including cranial nerve, cerebellar, and gait exam in a patient with ataxia and stiff person symptoms. Saccadic overshoot can be due to the cerebellar effects of the disease process [14]. Various ophthalmic manifestations of aGAS including vertical nystagmus [15], opsoclonus [14-16], and defects in smooth pursuit [16] can be seen.

The results of the anti-GAD test are essential as quantitative data (in our case anti-GAD>250 IU/Ml). Even low anti-GAD levels can present with clinical features like upbeat nystagmus (<30 IU/Ml) and type 1 DM (<100 IU/Ml) [12]. The anti-GAD titers are expected to be high for stiff person syndrome (SPS) or for cerebellar ataxia (CA) [17]. After diagnoses of most autoimmune CAs, a paraneoplastic origin must be ruled out, especially for the patients with subacute symptom onset. But for the cases with anti-GAD associated CA, this may not be necessary as concurrent malignancy is rare [18]. Antibodies to gliadin and transglutaminase need to be checked as there is an overlap between gluten ataxia and aGAS ataxia. A strict gluten diet may improve the ataxia in this subgroup regardless of detectable enteropathy [10]. Brain imaging can be helpful to differentiate subacute vs. chronic disease as the latter one can present with cerebellar atrophy on an MRI brain scan.

Given the postulation that this is strictly an autoimmune-mediated disease, treatment tends to consist of immune modulating therapies [19]. Despite given developing literature, there is no standard of care when it comes to the treatment of anti-GAD cerebellar ataxia. Treatment typically consists of glucocorticoids, IVIG, and plasmapheresis [20]. There is limited data regarding the time course of such treatments and if combining the treatments is of any benefit. Corticosteroids have been tried as the first-line therapy for anti-GAD associated neurologic disorders [20]. Additional case reports suggest that a combination of IVIG and delayed plasmapheresis show efficacy regarding symptomatic improvement [12, 19]. For maintenance therapy, it has been recommended that oral steroids, IVIG, azathioprine, and mycophenolate mofetil may be reasonable options [20]. Data also suggests that these treatments are more beneficial in acute/subacute presentations of anti-GAD cerebellar ataxia [11]. If the treatment is not providing symptomatic relief, an alternative treatment method should be tried. For our patient, we chose initially to treat with five days of high-dose IV steroids followed by a five-day course of IVIG. This regimen had resulted in a significant improvement in the patient’s symptoms and SARA score by day five of the IVIG course. There are other cases in the literature that show that the steroid and IVIG combination was used with variable outcomes [19].

The clinical course, prognosis, and the response to therapy are different in various patients. Age less than 60 years, subacute presentation, the absence of atrophy on brain imaging, and low anti-GAD titers were predictive of good outcomes [4].

The limitations of our study include that we did not check anti-GAD index, which is a ratio test between blood and CSF [17]. This test would have confirmed the intrathecal vs. systemic production of antibodies. We also did not repeat the anti-GAD antibody test during follow up visits, which would have helped to see the response of the antibody to the treatment [13].

## Conclusions

We presented a case with anti-GAD ab positive cerebellar ataxia, which we treated with a combination of a five-day IV steroid course immediately followed by IVIG treatment. This treatment regimen resulted in a significant improvement in the patient’s symptoms and functionality. More studies are needed to determine if the treatment regimen we chose will be as effective in other patients with the same condition. 

## Appendices

**Table 1:** Blood Workup pg: picograms; ml: milliliters; U: unit; IU: international units; mmol: millimoles; mg: milligrams; L: liter; dL: deciliters; ug: micrograms; mm: millimeters; ng: nanograms

Test	Value	Reference Range and Units
Vitamin b12	727	211 - 911 pg/mL
Rapid plasma reagin (RPR)	Nonreactive	Nonreactive
Angiotensin-converting enzyme level	20	9 - 67 U/L
Glutamic acid decarboxylase	>250	<5 [IU]/mL
Lactate	0.7	0.5 - 2.2 mmol/L
Pyruvate	0.88	0.30 - 1.50 mg/dL
Vitamin E level	17.6	5.7 - 19.9 mg/L
Ceruloplasmin	20	18 - 36 mg/dL
Copper	86	70 - 175 ug/dL
Human Immune Deficiency Virus (HIV) 1 and 2	Nonreactive	Nonreactive
Erythrocyte sedimentation rate (ESR)	6	0 - 15 mm/h
C-reactive protein (CRP)	<0.03	<0.50 mg/dL
Hemoglobin A1c	5.9	<5.7 %
Thyroid stimulating hormone (TSH)	1.26	0.27 - 4.20 u[IU]/mL
Prostate specific antigen (PSA)	0.14	0.0 - 4.0 ng/mL

**Table 2:** Antibody Screening Please note high anti-glutamic acid decarboxylase antibody titers. IU: International unit, mL: milliliters,  mg: milligram, dL: deciliters, index val: index value

Test	Value	Reference Range and Units
Rheumatologic antibodies	Negative	Negative
Thyroid peroxidase (TPO) Ab	403	<9 IU/mL
Immunoglobulin A	200	80 - 463 mg/dL
Antineutrophil cytoplasmic antibodies	Negative	Negative
Myeloperoxidase antibodies	Negative	Negative
Proteinase 3b antibodies	Negative	Negative
Anti-Sjögren's-syndrome-related antigen A (SSA) (RO) antibody	<1.0 negative	<1.0 negative [index_val]
Anti-Sjögren's-syndrome-related antigen B (SSB) (LA) antibody	<1.0 negative	<1.0 negative [index_val]
Tissue transglutaminase antibody b, immunoglobulin A	1	<4 [index_val]
Lyme immunoglobulin G/Immunoglobulin M antibody	<0.9	0.19 [index_val]
Neuronal nuclear antibody (Ri) Hu and Yo, CSF	Negative	Negative
Anti Ma and TA	Negative	Negative
Anti glutamic acid decarboxylase antibody	250.0	0.0-5 IU/mL

**Table 3:** Imaging Studies MRI: magnetic resonance imaging, CT: computed tomography, US: ultrasonography

Imaging Modality	Impression	
MRI brain	Unremarkable contrast-enhanced MRI scan of the brain	
CT chest abdomen pelvis	No primary malignancy or metastatic disease identified in the chest, abdomen, and pelvis	
US testis	1. Calcifications in the scrotum likely representing scrotal pearls, the largest one in the right scrotal measuring approximately 0.4 cm	
	2. The remainder of the examination is unremarkable	

**Table 4:** CSF Analysis CSF: cerebrospinal fluid; pg: picograms; ml: milliliters; U: unit; IU: international units; mmol: millimoles; mg: milligrams; L: liter; dL: deciliters; ug: micrograms; mm: millimeters; ng: nanograms

Test	Value	Reference Range and Units
CSF appearance		CLEAR AND COLORLESS
CSF nucleated cells	0 - 5 /uL	0
CSF red blood cells	<1 /uL	13
CSF corrected nucleated cells	0 - 5 /uL	<1
CSF glucose	40 - 75 mg/dL	68
CSF lactate dehydrogenase	U/L	23
CSF protein	15 - 45 mg/dL	47
CSF The Venereal Disease Research Laboratory test (VDRL)	Nonreactive	Nonreactive
CSF angiotensin-converting enzyme	<=15 U/L	8
CSF cytology	Negative for malignant cells	

**Table 5:** Iron Panel dL: deciliters; ug: micrograms; mm: millimeters; ng: nanograms

Test	Value	Reference Range and Units
Ferritin	78	15 - 400 ng/mL
Iron	67	42 - 135 ug/dL
Iron binding capacity	271	225 - 425 ug/dL
% transferrin saturation	25	15 - 50%

**Table 6:** Heavy Metal Screening dL: deciliters; ug: micrograms; mm: millimeters

Test	Value	Reference Range and Units
Blood arsenic	<3	<23 ug/L
Blood lead	<1	<5 ug/dL
Blood mercury	<4	<=10 ug/L
Blood manganese	1.1	<1.2 ug/L
